# Pre-Vitrification and Post-Warming Variables of Vitrified-Warmed Blastocysts That Are Predictable for Implantation

**DOI:** 10.3390/jcm12196389

**Published:** 2023-10-06

**Authors:** Anette Gabrielsen, Lea Hedegaard Iversen, Jens Fedder, Tilde Veng Eskildsen, Anne Lis Englund, Stine Ravn Hansen, Philippe Pinton

**Affiliations:** 1 Fertility Clinic, Horsens Regional Hospital, 8700 Horsens, Denmark; leaive@rm.dk; 2 Centre of Andrology & Fertility Clinic, Odense University Hospital, University of Southern Denmark, 5000 Odense, Denmark; fedder@dadlnet.dk (J.F.); tilde.veng.eskildsen@rsyd.dk (T.V.E.); 3 Fertility Clinic, Sealand University Hospital, 4600 Koege, Denmark; aleg@regionsjaelland.dk (A.L.E.); srhs@regionsjaelland.dk (S.R.H.); 4 Clinical and Translational Sciences, Ferring Pharmaceuticals, 2770 Kastrup, Denmark; philippe.pinton@ferring.com

**Keywords:** blastocyst, transfer, vitrification, warming, time-lapse, morphology, morphokinetics, prognostic factors, implantation, pregnancy

## Abstract

Human IVF embryos that are not used for fresh transfer are cryopreserved by vitrification for later embryo transfers. This study evaluates pre-vitrification and post-warming embryo characteristics that are suitable to predict the chance of clinical pregnancy in single vitrified blastocyst transfer (SVBT) cycles. In a multicenter observational trial (IMBOS trial), embryos were cultured in a time-lapse system before and after vitrification. Associations between clinical pregnancy, morphokinetic parameters, blastocyst collapse, KIDScore D5, pre-vitrification and post-warming Gardner scores, post-warming blastocyst size and re-expansion rates before SVBT were analyzed in 182 SVBTs which resulted in 89 clinical pregnancies. No association was found between clinical pregnancy after SVBT and the number of collapses or the maximal collapse size before vitrification. The multifactorial analysis of pre-vitrification Gardner scores showed a significant association with clinical pregnancy for trophectoderm grading but not for expansion/hatching status and inner cell mass grading. A significant association with clinical pregnancy was found for the time to reach a blastocyst after pronuclear fading (tB-tPNf), KIDScore D5 and post-warming size but not the rate of expansion or maximal expansion size. The selection of blastocysts for SVBT could benefit from using pre-vitrification parameters like tB-tPNf, trophectoderm grading and post-warming blastocyst size.

## 1. Introduction

Cryopreservation with vitrification technology is currently so effective that almost all thawed vitrified blastocysts survive and have the potential to achieve high pregnancy and live birth rates [1,2]. An increasing number of blastocysts are vitrified after IVF/ICSI, which is further supported by stricter policies for performing elective single embryo transfer (e-SET). Consequently, the number of ART treatment cycles involving frozen embryo replacement (FER) has increased. The FER treatment is beneficial in that it reduces or eliminates the risk of ovarian hyperstimulation syndrome [3,4]. Several studies indicate that it seems to provide better obstetric and perinatal outcomes [3,5], along with an increased cumulative pregnancy rate [6]. However, a recent meta-analysis warned against the unnecessary freezing and excessive use of a ‘freeze-all’ strategy because of the potentially negative impact on perinatal outcomes [7].

Blastocyst morphology is the primary selection criterion for vitrification; however, multiple blastocysts undergo morphological changes—like blastocyst collapse and others—which make the evaluation of blastocyst quality difficult [8]. Gardner and Schoolcraft developed a blastocyst scoring system in relation to blastocyst expansion, inner cell mass (ICM) and trophectoderm (TE) [9], yet it can be challenging to evaluate the quality of a vitrified-warmed blastocyst compared to a fresh blastocyst since the vitrification/warming process can cause additional morphological changes.

The subsequent culture of the warmed blastocyst provides a chance to evaluate the blastocoele re-expansion that occurs after warming. Several articles have been published on the correlation between blastocoele re-expansion speed, the morphological score of the blastocyst and the pregnancy outcome of single vitrified-warmed blastocyst transfers (SVBTs) using culture in standard incubators [10,11,12]. The post-warming re-expansion rate and cell survival percentage are considered important when making a clinical decision on whether to transfer a warmed blastocyst/s or warm additional embryos. Blastocoele re-expansion has been reported to have some predictive values for the implantation of vitrify-warmed blastocysts [12,13]; however, published data are contradictory. While some studies suggest that blastocysts at earlier stages of development and with lower expansion grades have better survival, implantation and live birth rates [3,5,6,14], other studies have conversely reported that blastocysts with higher expansion grades have better chances of implantation and live birth [8]. It has also been suggested that blastocysts that quickly re-expand after warming should be prioritized for transfer [13,15].

The introduction of time-lapse technology into the IVF laboratory has provided an important non-invasive tool for deselecting embryos that have not developed optimally and for identifying a cohort of embryos with a higher probability of resulting in the birth of a healthy child. Several publications on the selection of best-quality embryos via time-lapse vs. standard morphology exist [16,17,18,19,20]. Time-lapse monitoring showed that the number of blastocyst collapses is strongly associated with implantation potential [21,22]. Blastocyst collapse, expansion and re-expansion can only be quantified in cycles with fresh or frozen blastocysts using time-lapse recordings and not via culture in standard incubators [21,22,23]. Consequently, several studies have investigated the use of time-lapse incubation for the study of post-warming parameters [24,25,26]; however, few, if any, have used time-lapse to study the combined use of pre-vitrification and post-warming parameters [27].

In view of SVBT, the identification of pre-vitrification parameters can support the decision of which embryo should be warmed first for transfer. In addition, post-warming parameters can help to decide if another embryo should be warmed in case the first embryo does not develop properly. The aim of this study was to identify morphokinetic and morphological variables during embryo development before vitrification and after warming, which are suitable for predicting the chance of clinical pregnancy in SVBT cycles.

## 2. Material and Methods

### 2.1. Study Design

This study was performed as a non-interventional multi-center observational study conducted at three sites in Denmark. The primary objective was to evaluate the impact of vitrify-warmed blastocyst morphological development and blastocoele re-expansion on clinical pregnancy rates. The primary endpoint was the clinical pregnancy rate (defined as at least one intrauterine gestational sac with a fetal heartbeat at Week 7) from vitrify-warmed blastocysts.

The secondary endpoints were differences between morphokinetic timings (based on the assessment of tPNf, t2, t3, t4, t5, t6, t7, t8, tSB, tB, tEB, tHB), the number of collapses and collapse sizes during blastocyst development, Gardner and Schoolcraft [9] and KIDSCore D5v2 grading between 115 h and 120 h after insemination, survival rate after vitrification-warming, the measurement of expansion rate and size after warming until the blastocyst completely re-expands or until cryotransfer. Survival rate was calculated as the % of blastocysts that showed re-expansion after warming.

This study aimed at including 1200–1500 treatment cycles, which was estimated to be sufficient for observing at least one SVBT in at least 616 subjects. Enrolment started in December 2018, and the first oocyte pick-up (OPU) was performed in January 2019. The study was prematurely stopped on 31 December 2020 due to COVID-19 and the unlikeliness of reaching the aimed number of transfers within a reasonable time frame. This study was registered at ClinicalTrials.gov (NCT03697031).

### 2.2. Patients

Patients who underwent an elective single embryo transfer (e-SET) in an FER treatment cycle with vitrify-warmed blastocysts were eligible for inclusion. Either IVF or ICSI were accepted. Patients with contraindications for IVF or ICSI (patients with cancer and, e.g., estrogen receptor-positive mamma cancer; severe cardiovascular disease; severe genital abnormalities including “hidden” ovaries), as well as oocyte donation cycles, were excluded from participation.

### 2.3. Hormone Stimulation

Controlled ovarian stimulation (COS) was performed with follitropin delta (Rekovelle^®^, Ferring Pharmaceuticals, Kastrup, Denmark) using an individualized dosing regimen based on the AMH and body weight of each patient [28].

### 2.4. Laboratory Procedures for Fresh Cycles

Standard procedures according to established clinical practice at each participating site were applied for the aspiration and washing of cumulus–oocyte complexes (COC). Semen was collected via orthograde ejaculation (*n* = 320). In a few cases, donor sperm was used (*n* = 29). Sperm preparation was conducted at all sites by density gradient centrifugation using a 40%/80% gradient (Ref 84000060, Origio, Måløv, Denmark) prepared with Sperm wash (Ref 84050060, Origio). Sperm pellets were dissolved in a fertilization medium (Sequential Fert, Ref 83010010A, Origio) prior to further use. The choice of a fertilization method depended strictly on the patient’s diagnosis and previous fertility treatment and history. IVF and ICSI procedures were performed according to each clinic’s standard procedure.

Oocytes that were inseminated using ICSI were immediately transferred into a pre-equilibrated EmbryoSlide (Vitrolife AB, Gothenborg, Sweden) and placed in the Embryoscope time-lapse system (Vitrolife A/S, Viby J, Denmark). In the case of insemination by IVF, zygotes were placed in the EmbryoScope on day 1 after insemination. Embryo culture was performed until day 5 for 115–120 h after insemination using 6% CO_2_, 5% O_2_ and 94% N_2_ at 37.0 °C. On day 5, blastocysts were either used for fresh transfer or were vitrified. Additional treatments like preimplantation genetic testing for aneuploidy (PGT-A) and assisted hatching were not performed. All three clinics used the same continuous single culture media system (SAGE-1, Ref 67010010A, Origio, Måløv, Denmark) and oil (Liquid Paraffin, Ref 10100060A, Origio) with no media change during culture up to day 5. All embryo transfers were performed according to the standard procedure at each clinic.

### 2.5. Vitrification/Warming Procedure

The vitrification and warming of blastocysts were performed with a closed vitrification system using RapidVit^TM^ Blast/RapidWarm^TM^ Blast (Ref. 10119/Ref. 10120, Vitrolife) as vitrification/warming media and a closed system, Rapid-I, as the carrier (Vitrolife) according to the manufacturer’s instructions. Warming blastocysts with the best morphology, as judged by Gardner criteria, were primarily selected. Immediately after warming, vitrified-warmed blastocysts were placed in the EmbryoScope. The typical time from starting the warming process to placement in the EmbryoScope took 10 to 15 min, and the post-warming culture period ranged from three to four hours.

### 2.6. Endometrial Preparation for Cryotransfer

In women with a regular menstrual cycle, cryotransfer was performed in an unstimulated cycle, and the hCG trigger was used when the leading follicle was 18 mm. In women with irregular cycles or with a poorly developed endometrium in an unstimulated cycle, orally administered estrogen was used from the 2nd to 3rd cycle day. A vaginal progesterone supplement was started once the endometrium reached 8 mm, and cryo transfer was scheduled 6–7 days later. 

### 2.7. Parameters Assessed by Time-Lapse in Fresh Cycles

Parameters assessed during embryo development before vitrification were morphokinetic timings (tPNf, t2, t3, t4, t5, t6, t7, t8, tSB, tB, tEB, tHB) [19], the number of blastocyst collapses and collapse size, Gardner and Schoolcraft grading at 115 h (±1 h) post insemination and KIDSCore D5v2 (Vitrolife) grading between 115 h and 120 h post insemination. Evaluations that included morphokinetics, like tB-tPNf, were related to the time of pronuclear fading (tPNf) in order to enable a comparison of IVF and ICSI embryos.

Collapse was defined as any event where all trophectoderm cells separated from the zona pelllucida. The maximal collapse size was defined as the maximal collapse size in percentage for all collapses that the blastocyst had during embryo culture (100 × [(size before collapse − size at maximum collapse)/size before collapse]). In the case of no collapses, the maximal collapse size was set to 0.

### 2.8. Parameters Assessed by Time-Lapse after Vitrification/Warming

Parameters assessed after vitrification-warming were survival rate, size after warming and expansion rate immediately after warming until the time the blastocyst had completely re-expanded or experienced cryotransfer, and Gardner and Schoolcraft blastocyst grading was assessed immediately before SVBT. Vitrify-warmed blastocysts were placed in the EmbryoScope immediately after warming. The time when the first image emerged on the EmbryoViewer was designated as Time 0. The first measurement of the size after warming was performed at Time 0, with subsequent measurements made every 20–30 min until the blastocyst had either started to hatch or were removed from time-lapse incubation for transfer.

The re-expansion rate during post-warmed culturing was conducted by measuring the surface area of the blastocyst every 20–30 min. Re-expansion was evaluated by estimating the slope (b) of the log(blastocyst size) versus the time curve using linear regression. The exponential rate of re-expansion (as percentage growth per time unit) was calculated from the estimated slope as $100\cdot \left(e^{b}-1\right).$ Measurements for all blastocysts from the three participating clinics were performed using the same embryologist for all three sites. All size measurements (collapse and expansion size pre-vitrification and size post-warming as area in µm^2^) were performed using the EmbryoViewer Ellipse Tool, as previously described [29].

### 2.9. Data Collection and Statistical Analysis

Data were collected via the clinics’ electronic medical record system (FORMATEX) hosted by the Dansk Medicinsk Data Center and electronically via the EmbryoViewer database. Timings (tPNf, etc.), collapses, size measurements and KIDScore were only captured in the database for embryos that were transferred in either a fresh or SVBT cycle. Gardner and Schoolcraft’s grading was captured for all cryopreserved blastocysts. For evaluation, respective data points from the two databases were exported into Microsoft Excel, merged and de-identified.

The primary endpoint was the clinical pregnancy rate from vitrify-warmed blastocysts. The association between the various embryological parameters and the probability of a positive clinical pregnancy was investigated using logistic regression models where continuous embryological parameters were entered as continuous covariates and discrete embryological parameters were entered as discrete factors. For the analyses of cryotransfers, where a subject could contribute with data from repeated cycles, a mixed model was used where the log odds for different subjects were assumed to be normally distributed. For continuous embryology parameters, the fitted model was illustrated together with data in a figure, and the estimated slope of the covariate and the *p*-value for the test of the covariate, which was zero, were displayed. For discrete embryology parameters, the estimated rates were calculated for each category together with the *p*-value for the test of all categories, which were equal.

For re-expansion parameters and Gardner and Schoolcraft blastocyst grading, multifactorial models were used to investigate the influence of the speed, initial size and maximal size, and the expansion and hatching status, inner cell mass and trophectoderm, respectively. The models used in these analyses were the same as the single factor/covariate models.

Clinical pregnancy rates per transfer after cryopreserved transfers were compared using a mixed-effect logistic regression model where the log odds for different subjects were assumed to be normally distributed. From this model, the expected clinical pregnancy rate per transfer was estimated, and the *p*-value for the two rates being equal was calculated.

Gardner and Schoolcraft blastocyst grading, the measurement of expansion size, the number of collapses, the measurement of collapse size and registration of KIDScore D5 v.2 were also used as covariates or factors in the analysis and association with the primary endpoint.

All statistical analyses were performed using the SAS software (SAS Institute Inc., version 9.4M6, Cary, NC, USA).

## 3. Results

### 3.1. Patient Demographics and Clinical Outcome Analysis

The demographics and baseline characteristics of the study population are presented in Table 1. The characteristics of the subset of women with SVBT were similar to all women included in the trial. On average, women were young (~30 years), and the majority suffered from primary infertility, whereas the most common cause of infertility was the male factor (in 40% of couples).

In total, 349 women started ovarian stimulation, but only 271 women had fresh or frozen embryo transfer, partly due to temporary clinic closures due to COVID-19 pandemic restrictions. There was a total of 377 embryo transfers in 271 subjects; 195 were fresh transfers, where 99 resulted in a clinical pregnancy (50.8%).

Regarding cryotransfers, a total of 201 blastocysts were warmed, of which 182 were transferred in a cryotransfer, which gave a usability rate of 91%. For 7 subjects, one blastocyst was warmed but not transferred, 10 subjects had two blastocysts warmed and one transferred, and 1 subject had three blastocysts warmed and one transferred.

The 182 cryotransfers were performed in 130 women and resulted in 89 clinical pregnancies (48.9%). Artificial laser collapse before vitrification was performed in 89/182 blastocysts (49%), of which 45 ended with a clinical pregnancy (50.6%). Of the 93 blastocysts without laser collapse, 44 ended with a clinical pregnancy (47.3%). This indicates that artificial laser collapse did not have a significant effect on clinical outcomes.

After adjusting for repeated transfers within the same women, the estimated clinical pregnancy rates per transfer were 50.9% for fresh transfers and 54.6% for cryotransfers. There was no statistically significant difference in the rates per transfer between fresh and cryopreserved transfers (*p* = 0.47). A clinical pregnancy rate of 52% was achieved for woman who started stimulation. The study was stopped before all frozen blastocysts from the stimulation cycles were warmed and had the opportunity to be transferred.

These experimental results allowed us to conclude that the clinical outcome after SVBT provided a sufficiently high success rate for the identification of parameters and selection of blastocysts for warming and subsequent cryo-transfer.

### 3.2. Probability of Clinical Pregnancy with Gardner and Schoolcraft Grading before Vitrification and before Cryotransfer

Comparing Gardner and Schoolcraft blastocyst grading at the time of vitrification and cryotransfer, identical scores for 21% of blastocysts were revealed. For most blastocysts, scores changed in both directions for all three parameters.

#### 3.2.1. Expansion and Hatching Status Grading

**Figure 1:**
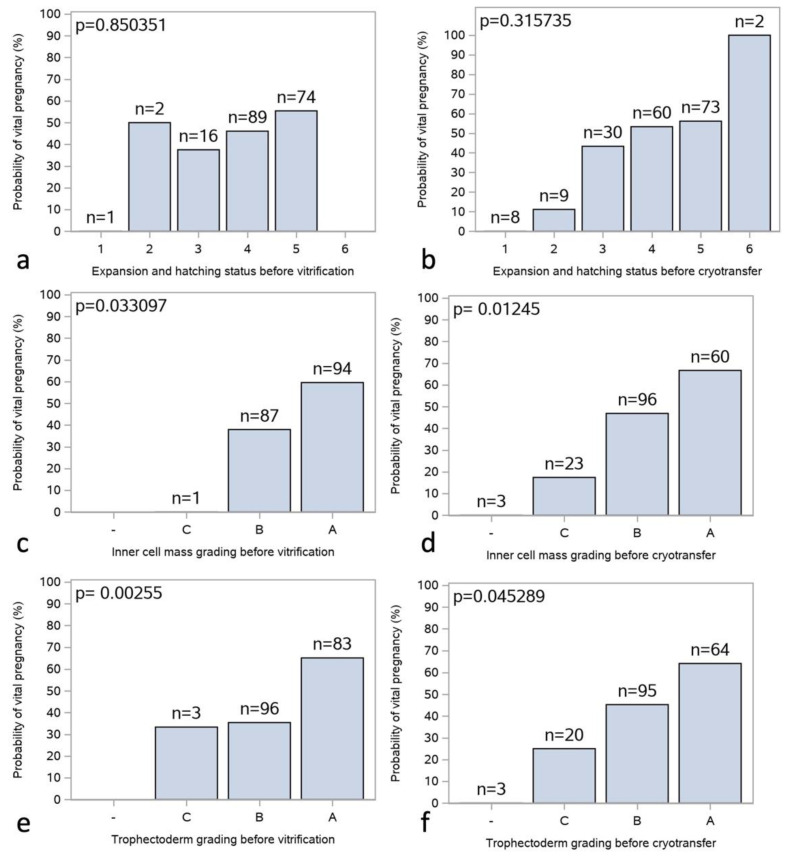
Correlation of Gardner and Schoolcraft score before vitrification and cryotransfer with a probability of a clinical pregnancy. The probability of clinical pregnancy in relation to the Gardner and Schoolcraft grading score is shown for 182 SVBT cycles in 130 subjects: (**a**,**b**) before vitrification and cryotransfer for expansion and hatching; (**c**,**d**) for ICM; (**e**,**f**) and trophectoderm, respectively. In univariate analysis, ICM and trophectoderm were significant before vitrification and cryotransfer, as indicated in the respective graph. ICM, inner cell mass; SVBT, single vitrified blastocyst transfer.

The association between expansion and hatching status and the probability of clinical pregnancy is illustrated in Figure 1a,b. Most embryos that were selected for cryotransfer had scores of either 4 or 5 before vitrification and scores of 3, 4 or 5 before cryotransfer. There was no statistically significant association between the expansion, hatching status or probability of clinical pregnancy.

#### 3.2.2. Inner Cell Mass Grading

The association between ICM grading and the probability of a clinical pregnancy is illustrated in Figure 1c,d. Most blastocysts had gradings of A or B, and there were clear trends of higher probability for clinical pregnancy with higher grades. There was a statistically significant association between ICM before vitrification as well as before cryotransfer.

#### 3.2.3. Trophectoderm Grading

The association between trophectoderm grading and the probability of a clinical pregnancy is illustrated in Figure 1e–f. There were clear trends of higher probability for clinical pregnancy with a higher trophectoderm grade. The association between trophectoderm grade and the probability of clinical pregnancy was statistically significant.

#### 3.2.4. Multifactorial Analysis

A multifactorial model was used to simultaneously investigate the association between expansion and hatching status, ICM grading, trophectoderm grading, and the probability of clinical pregnancy. The results are summarized in Table 2 and show that trophectoderm grading was the only factor that was statistically significant before vitrification but not before cryotransfer. The lack of significance for trophectoderm grading before cryotransfer was probably due to strong confounding with inner cell mass grading, which had a stronger association with clinical pregnancy prior to cryotransfer.

These data allow us to conclude that the trophectoderm grade is the only pre-vitrification parameter of the Gardner and Schoolcraft blastocyst grading system that might offer a positive selection of which blastocyst to warm first.

### 3.3. Blastocyst Collapse before Vitrification

**Figure 2:**
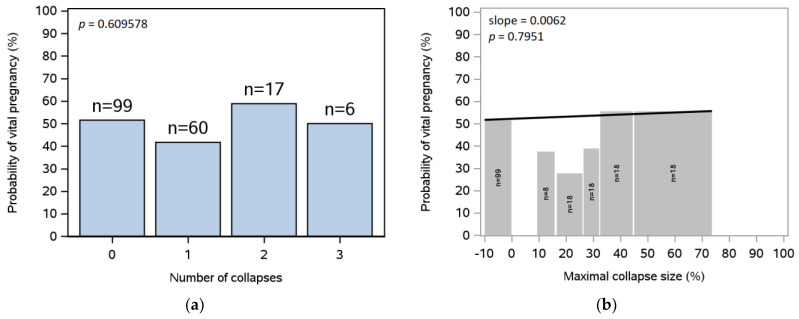
Probability of clinical pregnancy with number of collapses and maximal collapse size before vitrification. (**a**) The probability of clinical pregnancy in relation to the number of collapses during culture before vitrification (from 182 SVBT cycles in 129 subjects); (**b**) The association between the probability of clinical pregnancy and maximal collapse size as a percentage (from 179 SVBT cycles in 128 subjects) is based on the statistical analysis with slope (black line) and *p*-value indicated; neither were statistically significant. SVBT, single vitrified blastocyst transfer.

The association between the number of collapses during embryo culture before vitrification and the probability of a clinical pregnancy following SVBT is shown in Figure 2a. Most embryos had zero or one collapse, and there was no statistically significant association between the number of collapses and the probability of clinical pregnancy. Figure 2b shows the association between maximal collapse size (calculated for each blastocyst as the maximal relative change in blastocyst size across collapses and set to zero for a blastocyst without collapses) and the probability of a clinical pregnancy. No association was observed.

### 3.4. Blastocyst Size after Warming, Maximal Re-Expansion and Exponential Rate of Re-Expansion

**Figure 3:**
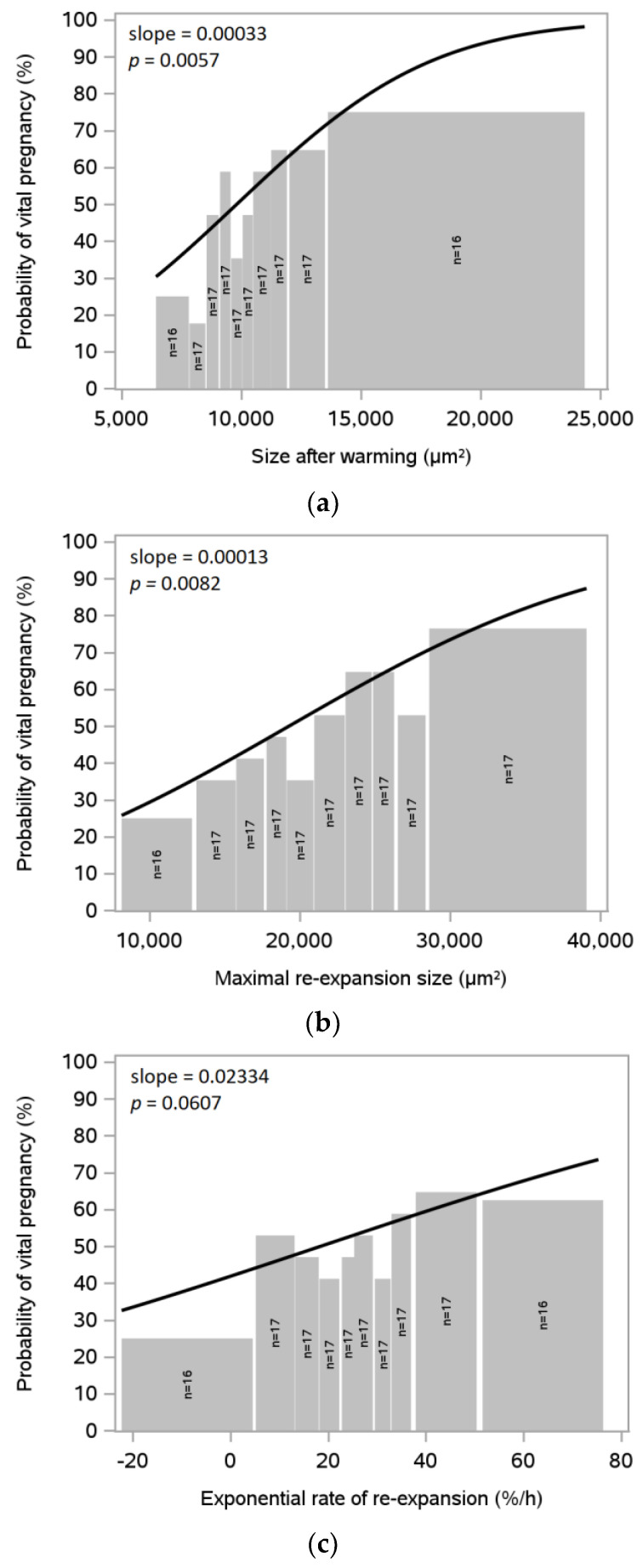
Probability of clinical pregnancy with blastocyst size after warming, maximal re-expansion and exponential rate of re-expansion. (**a**) Data on blastocyst size after warming in µm^2^; (**b**) Maximal re-expansion size in µm^2^; and (**c**) Exponential rate of re-expansion in %. For each graph, the datasets were grouped in approximately 10 equal parts with the number of cycles indicated for each bar. In each graph, the curve shows the estimated probabilities for pregnancy based on statistical analysis with the slope gradient (regression line) and *p*-value indicated. SVBT, single vitrified blastocyst transfer.

The blastocyst size after warming was positively associated with the probability of clinical pregnancy (*p* = 0.0057; Figure 3a). The maximal blastocyst re-expansion size after warming showed a positive and significant association with the clinical pregnancy rate (*p* = 0.0082; Figure 3b). Figure 3c shows the association between the rate of re-expansion where a regression was performed on log(size) vs. time, assuming an exponential growth, and the rate on the log scale $\left(b\right)$ was converted to a percentage of growth as $100\cdot \left(e^{b}-1\right)$.

To investigate which re-expansion endpoint had the strongest association with clinical pregnancy rate, a multifactorial regression model was used with size after warming, including the maximal re-expansion size and rate of re-expansion as covariates. In this analysis, the size after warming was the only factor that was statistically significant (*p* = 0.0257). This indicates that the blastocyst size after warming alone might explain the positive association between re-expansion parameters and clinical pregnancy.

### 3.5. Artificial Laser Collapse before Vitrification and Post-Warm Parameters

The use of artificial laser collapse resulted in a more consistent and limited but not significant size after warming (*p* = 0.0731) and significantly larger maximal re-expansion size (*p* = 0.0365). However, the probability for clinical pregnancy for size after warming, including maximal re-expansion size and the exponential rate of re-expansion, was not significant in cryotransfers with or without artificial laser collapse before vitrification (*p* = 0.1046 versus 0.2848, *p* = 0.2645 versus 0.1009 and *p* = 0.2663 versus 0.2422, respectively). Based on these data, we concluded that artificial laser collapse did not result in bias.

### 3.6. Expansion during Culture before Vitrification

**Figure 4:**
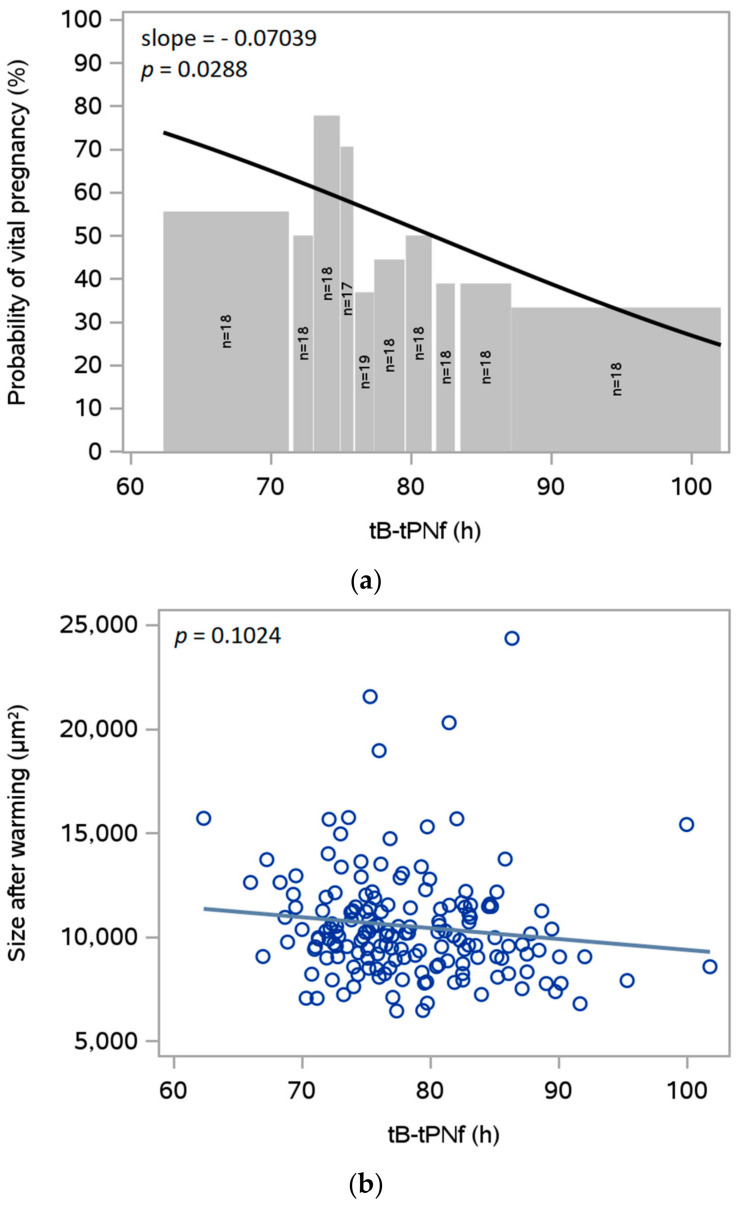

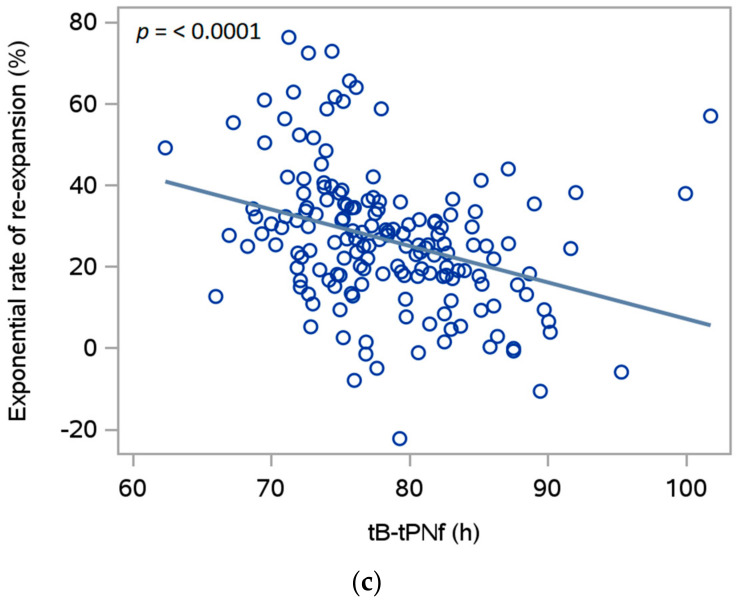
The probability of clinical pregnancy with tB-tPNf during culture and the association of tB-tPNf with size after warming and the exponential rate of re-expansion. (**a**) Data on development from the time of pronuclear fading to time of full blastocyst (tB-tPNf) from 180 SVBT cycles in 128 subjects were grouped in approximately 10 equal parts with the number of cycles indicated for each bar. The estimated probability for clinical (vital) pregnancy is based on the statistical analysis with slope and *p*-value indicated. The association between (**b**) tB-tPNf and size after warming and (**c**) the exponential rate of re-expansions are shown with the corresponding *p*-value and slope gradient (regression line).

The expansion of the blastocyst during embryo culture was not measured directly. As an indicator of expansion, differences between morphokinetic timepoints were investigated, i.e., tB-tSB, tB-tPNf, tB-t8 and tB-t5. Of these, a significant association with the probability of clinical pregnancy after SVBT was found for tB-tPNf (*p* = 0.0288; Figure 4a). There was also an association between tB-tPNf and the blastocyst size after warming (Figure 4b), as well as the rate of re-expansion (Figure 4c). This association was statistically significant for the rate of re-expansion but not for the size after warming.

tB-tPNf can be considered a kinetic factor that depicts the speed of growth of an embryo. As the definition of tB includes that the blastocyst has filled the perivitelline space, it is also an indicator of early expansion. Experimental data allow us to conclude that a blastocyst with a fast growth characteristic before vitrification may also continue to grow fast after warming, expressed by faster re-expansion compared to others.

### 3.7. KIDScore before Vitrification

A positive and statistically significant association between KIDScore D5 v.2 and the probability of clinical pregnancy was found for both fresh transfers (*p* = 0.0036) and cryotransfers (*p* = 0.0008). The mean score of SVBT was 7.37 (minimum 0, maximum 9.8).

KIDScore D5 v.2 is a general quality marker for human embryos that have reached the blastocyst stage. Although this algorithm was developed on data from fresh embryo transfer cycles, it is equally applicable to cryotransfers.

## 4. Discussion

This study evaluated the effect of pre-vitrification and post-warming parameters for clinical outcomes in SVBT using time-lapse monitoring. The expansion during embryo culture (tB-tPNf), the trophectoderm score before vitrification, blastocyst size after warming and scoring using a universal morphokinetic algorithm were the most important prognostic parameters for predicting clinical pregnancy. By contrast, the number of blastocyst collapses before vitrification and re-expansion rate and maximal re-expansion size after warming showed less of a clear association regarding their prognostic value for predicting a clinical pregnancy outcome.

Several studies have investigated factors that can be used to predict the potential of an embryo after vitrification warming using either a conventional or time-lapse culture [8,10,11,13,14]; however, the findings reported after conventional culture for the degree of expansion at the blastocyst stage and the morphological quality of ICM and trophectoderm are sometimes contradictive. Some studies found the degree of expansion or blastocyst diameter before vitrification to be predictive but not morphology [10,11,30], whereas others reported the opposite [8,31,32]. Independent of using a time-lapse or conventional culture, morphological evaluations can be challenging due to inter- and intra-observer variations [33], and such variations have also been shown to influence the decision of whether to cryopreserve or discard an embryo [34]. Furthermore, blastocyst expansion status assessed during conventional culture is a qualitative parameter but not an adequate measure of the speed of embryo development. In the current study, we applied time-lapse monitoring, which is supposed to allow a more objective assessment with measurable parameters. A shorter time span to reach the full blastocyst stage after pronucleus fading (tB-tPNf), which is an expression of the speed of embryo development, was identified as a positive predictor for clinical pregnancy. On the other hand, the expansion and hatching stage, as defined by Gardner and Schoolcraft criteria, was not a predictor for clinical pregnancy. This may indicate morphological changes that may occur after initial blastocyst formation and during blastocyst expansion. One such potential factor is the occurrence of blastocyst collapses during expansion. Several studies have reported that the frequency of collapse negatively impacts clinical outcomes [22,35,36,37], although this was not confirmed by another study [14]. Our evaluation also did not find such a correlation; however, the amount of data for this feature may be too low in our study to draw a definite conclusion.

In general, it is obvious that findings from one study may be based on a different measure than those obtained from other studies, especially when different incubation conditions with or without time-lapse are used; therefore, a fair comparison may not be always possible.

Many studies have investigated post-warming parameters, and the degree of blastocyst re-expansion after warming has been reported as a positive predictor of clinical pregnancy by most studies [8,10,13,24,25,38] but not all [39]. However, the post-warming culture time (3–7 h), the mode of incubation (conventional versus time-lapse) and the definition of re-expansion (e.g., expansion status versus degree of re-expansion versus time taken for the blastocyst to fill the whole perivitelline space) differs between these studies. In our study, we made a quantitative measure of the exponential rate of re-expansion, based on the size of the blastocyst immediately after warming and before embryo transfer and the post-warming culture time period ranged from three to four hours. In the univariate analysis, this parameter looked promising, but multifactorial analysis showed that the probability of clinical pregnancy was mainly associated with the blastocyst size immediately after warming. Interestingly, two studies reported that the blastocyst area as a measure of size is also a potential predictive factor for clinical pregnancy in fresh transfer cycles [23,36], a feature that we have not evaluated in this study.

The potential benefit of size after warming has already been reported in a previous study that also investigated re-expansion based on changes in size after warming [26]. These authors conclude that the time interval between the completion of warming and the start of re-expansion has no effect on clinical outcomes, but blastocysts that did not initiate re-expansion had very poor clinical outcomes. Furthermore, the authors showed in a hierarchical model with defined cut-off values that the implantation rate was positively correlated with the initial area and the maximum size that was reached after warming. Our findings are in line with the results of this study for the initial size after warming but not for maximum re-expansion size.

A recent study reported that the quantitative change in the blastocyst diameter between warming and 2 h post-warming incubation correlated with clinical pregnancy in 115 SVBT cycles [27]. This result was based on univariate analysis—which is in line with our findings—but was subsequently not confirmed by multivariate analysis. In general, this implies that more studies and data are needed for further clarification.

Another study that also used a post-warming time-lapse assessment was published by Kovačič et al. [40]. These authors presented a very detailed and minute analysis of the post-warming re-expansion of blastocysts that artificially collapsed before vitrification, using a timeframe of five minutes between one measurement and the following. They identified four highly dynamic re-expansion patterns. However, these authors could not find a correlation with live birth due to a small sample size. In our study, the timeframe for assessment was 20 to 30 min, and post-warming development was best described by a linear model. Due to the larger time window for assessment, there were smaller contractions with the pulsing of blastocysts, as reported by Kovačič et al. [40] and, thus, the detection of different re-expansion patterns might not be possible with our approach.

In approximately half of the SVBTs, we applied artificial laser collapse before vitrification; however, we found no significant difference between both groups regarding their size after warming, and like Kovačič et al. [40], we noted no difference in the probability for clinical pregnancy. More compact blastocysts were considered to better survive the osmotic stress of vitrification-warming compared to those showing a visible blastocoel after warming (as discussed in [40]). However, our assumption that the probability of clinical pregnancy increases with a larger size immediately after warming, including blastocysts with a visible blastocoel, does not support this. Nevertheless, the protocols used for vitrification-warming include a variety of media, materials, handling steps and timings, which could all influence size after warming, re-expansion and clinical outcomes. This illustrates that only studies with a comparable set-up may give relevant answers in this complex discussion.

Although certain post-warming parameters are highly predictive for subsequent embryo transfer, such parameters may be of limited practical benefit. The warming of a second embryo is usually only performed if the first embryo does not survive or shows severe signs of degeneration. Warming a second embryo due to a potential prognostic parameter that turns out to be not optimal—like size after warming—may be less likely in clinical practice, despite the option of re-vitrification of one of the two embryos [41]. Still, the preferred option is to have parameters at hand that can be used to decide which embryo to warm to avoid any risk of potential embryo wastage.

This current study demonstrates that a combination of the speed of development and trophectoderm morphology in a fresh cycle is suitable for pregnancy prediction in a subsequent SVBT cycle. Such a combination of kinetic and morphological parameters is often applied in morphokinetic algorithms that have been developed using time-lapse technology for implantation prediction in fresh embryo transfer cycles [19,42]. In this study, we tested an algorithm that included parameters for up to day 5 of fresh embryo development prior to vitrification. Our results confirmed a significant association between the blastocyst grading scores and the probability of clinical pregnancy in SVBT cycles. Similar findings were recently reported for the same algorithm of SVBT in a minimal ovarian stimulation program [43], where it worked well for the prediction of pregnancy and live birth outcomes in maternal patients >35 years but to a lesser extent in younger patients. The same group of authors took this approach a step further, using a completely automatic model based on artificial intelligence (AI) and deep learning that did not require any morphological or morphokinetic embryo assessment by the embryologist [44]. When applied to SVBT cycles, the automatic model performed at least as well as standard morphological or morphokinetic assessments regarding pregnancy prediction. In view of these more recent developments, it may very well be that future AI-based models could be a better alternative for pregnancy prediction and deciding which embryo to warm first for SVBT.

Considering the strengths and weaknesses of this multicentric study, the study protocol was close to daily clinical practice in the three participating centers. All embryologists were aligned in time-lapse annotations; however, size measurements were performed by one embryologist alone for data derived from all three clinics. Our data were exclusively derived from the culture up to day 5 and, thus, for blastocysts that were vitrified on day 6 or day 7, other prognostic parameters than those described in this manuscript may be relevant [30]. Unfortunately, the study was prematurely terminated due to the COVID-19 pandemic. This has reduced the power of this study, and some findings that were not significant could potentially be relevant with larger numbers. A larger study cohort may even allow subgroup analysis according to the reason for infertility. IVF units that wish to test the findings of this study need to use time-lapse equipment for the evaluation of parameters like maximal re-expansion and re-expansion rate.

## 5. Conclusions

This study confirms that using pre-vitrification parameters like tB-tPNf and trophectoderm grading are predictive for clinical pregnancy and may help when selecting which blastocyst to use for SVBT. In addition, this study demonstrates that blastocyst size immediately assessed after warming constitutes a prognostic post-warming parameter that can be relatively and easily assessed and may not even require a time-lapse system. Also, the fact that this evaluation can be performed immediately after warming and, thus, at a very early point in time offers a clear advantage compared to late predictive parameters.

## Figures and Tables

**Table 1:** Demographics and baseline characteristics.

	Women with SVBT (*n* = 130)	All Women (*n* = 349)
Age, years		
Mean ± SD	30.3 ± 3.73	30.5 ± 3.75
AMH, pmol/L		
Mean ± SD	25.2 ± 16.9	23.4 ± 15.9
BMI, kg/m^2^		
Mean (SD)	24.5 ±3.54	24.4 ± 3.80
Body weight, kg		
Mean ± SD	69.0 ± 10.6	69.2 ± 11.3
Duration of infertility, months		
Mean ± SD	27.6 ± 14.0	29.2 ± 16.2
Any Previous Pregnancy, n (%)		
Yes	36 (28)	93 (27)
No	94 (72)	256 (73)
Any Previous Birth, *n* (%)		
Yes	9 (7)	19 (5)
No	121 (93)	330 (95)
Reason for infertility, *n* (%)		
Anovulation	2 (2)	6 (2)
Tubal	10 (8)	19 (5)
Uterine factor	1 (1)	6 (2)
Male infertility	54 (42)	139 (40)
Unexplained	20 (15)	57 (16)
Other	19 (15)	58 (17)
Missing	24 (18)	64 (18)

AMH, Anti-Mullerian hormone; BMI, body mass index; SVBT, single vitrified-warmed blastocyst transfer; SD, standard deviation.

**Table 2:** Multifactorial analysis of the association between Gardner and Schoolcraft grading and the probability of clinical pregnancy.

*p*-Value	Expansion and Hatching Status	Inner Cell Mass	Trophectoderm
Before vitrification	0.8432	0.3153	0.0247
Before cryotransfer	0.2815	0.0965	0.9763

## Data Availability

The datasets in this study are available from the corresponding authors on reasonable request.
